# Complications of pupil expansion devices: a large real-world study

**DOI:** 10.3389/fopht.2023.1283378

**Published:** 2023-12-12

**Authors:** Tal Yahalomi, Omar Elhaddad, Venkata Avadhanam, Derek Tole, Kieran Darcy, Eliya Levinger, Raimo Tuuminen, Asaf Achiron

**Affiliations:** ^1^ Department of Ophthalmology, Samson Assuta Ashdod Hospital, Faculty of Health Sciences, Ben-Gurion University of the Negev, Be’er Sheva, Israel; ^2^ Bristol Eye Hospital, University Hospitals Bristol NHS Foundation Trust, Bristol, United Kingdom; ^3^ Faculty of Medicine, Alexandria University, Alexandria, Egypt; ^4^ Ophthalmology Department, Soraski Medical Center, Sackler Faculty of Medicine, Tel-Aviv University, Tel Aviv, Israel; ^5^ Department of Ophthalmology, Kymenlaakso Central Hospital, Kotka, Finland; ^6^ Helsinki Retina Research Group, Faculty of Medicine, University of Helsinki, Helsinki, Finland

**Keywords:** cataract surgery, pupil expansion device, posterior capsular opacification, pseudophakic cystoid macular edema, uveitis

## Abstract

**Purpose:**

To assess the risk for uveitis, pseudophakic cystoid macular edema (PCME), and posterior capsular opacification (PCO) associated with the use of pupil expansion devices in cataract surgery.

**Design:**

A retrospective comparative cohort study.

**Participants:**

Patients who underwent routine cataract surgery with and without pupil expansion devices at the Department of Ophthalmology, Bristol Eye Hospital, UK, between January 2008 and December 2017.

**Methods:**

This study included 39,460 eyes operated without a pupil expansion device and 699 eyes operated with the device. Odds ratios for uveitis and PCME when using a pupil expansion device were calculated using univariate and multivariate regression analysis, having age, gender, diabetes, pseudoexfoliation, and pupil expansion device as independent variables. Multivariate Cox regression controlling for age and gender was used to estimate hazard ratios (HR) for Nd : YAG laser capsulotomies.

**Results:**

Postoperative uveitis and PCME were reported in 3.9% and 2.7% of the eyes operated with a pupil expansion device compared to 2.3% and 1.3% operated without the device (p=0.005 and p=0.002, respectively). In univariate regression analysis, eyes with pupil expansion devices showed a higher risk of postoperative uveitis or PMCE after cataract surgery (OR 1.88, 95%CI 1.39-2.55, p<0.001). In multivariate regression analysis, the risk for PMCE was greater among diabetic patients and in eyes with a pupil expansion device than in those without (OR 1.50, 95%CI 1.24-1.83, *P*<0.001; OR 1.90, 95%CI 1.16-3.11, *P*=0.01). In Cox regression analysis adjusted for the patient’s age and gender, the use of a pupil expansion device was associated with higher Nd : YAG laser capsulotomy rates (HR 1.316, 95%CI 1.011-1.714, P=0.041).

**Conclusion:**

In our large cohort study, the use of pupil expansion devices in cataract surgery was associated with an increased risk of major postoperative complications. Effective anti-inflammatory treatment and follow-up are warranted in eyes operated with a pupil expansion device.

## Highlights

- Ensuring adequate pupil dilation is fundamental for successful and safe phacoemulsification surgery and when pharmacological strategies fail to achieve sufficient mydriasis, mechanical pupil expansion techniques are required.
- This large real-world study of over 40,000 eyes demonstrated that eyes operated with pupil expansion devices had a higher risk for major postoperative complications after cataract surgery, namely uveitis, PMCE, and PCO.
- The study demonstrates that using pupil expansion devices during cataract surgery may increase the risk for major postoperative complications and should be considered when planning the anti-inflammatory medication regime and follow-up after the surgery.

## Synopsis

A large real-world study of more than 40,000 eyes found that using pupil expansion devices increased the risk of uveitis, PMCE, and PCO. Careful consideration of anti-inflammatory medication and postoperative monitoring is advised.

## Introduction

Small pupils are a well-established risk factor for cataract surgery. In patients with inadequate pupil dilation, intraoperative complications such as vitreous loss and capsular rupture are more frequent (1). Poorly dilated pupils are related to local and systemic comorbidities, including diabetes, pseudoexfoliation syndrome (PXF), age-related degenerative changes in the iris, and intraoperative floppy iris syndrome (IFIS) (2).

Ensuring adequate pupil dilation is fundamental for successful and safe phacoemulsification surgery. When pharmacological strategies fail to achieve sufficient mydriasis, several efficient mechanical pupil expansion techniques are available, for instance, iris retractors, manual stretching with hooks, and other pupil expansion devices (3). The time required and the amount of iris trauma caused by these various techniques vary (4). In a retrospective study by Nderitu et al., higher rates of postoperative anterior uveitis and corneal edema were associated with the use of pupil expansion ring (5). In a *post hoc* analysis by Taipale et al., the use of a pupil expansion device predisposed eyes to aqueous flare, macular thickening, and increased risk of clinically significant pseudophakic cystoid macular edema (PCME) after otherwise uncomplicated cataract surgery (6).

Here, we aimed to assess by a large retrospective registry the real-world evidence whether pupil expansion devices are associated with the risk for major postoperative complications in cataract surgery, namely uveitis, PCME, and posterior capsular opacification.

## Methods

This was a registry-based retrospective cohort study of consecutive adult cataract surgeries performed at the Department of Ophthalmology, Bristol Eye Hospital, UK. Patients were enrolled between January 2008 and December 2017 and were admitted according to the national guidelines for managing cataracts. This study received the local ethics community approval (CORN/SE/2021-2022/02 and was presented to the local audit authority) and adhered to the tenets of the Declaration of Helsinki.

Inclusion criteria were all patients who underwent phacoemulsification surgery and intraocular lens implantation with or without a pupil expansion device. Those devices included Malyugin rings (MicroSurgical Technology/MST Inc., Redmond, WA), Morcher Pupil dilator Type 5S (FCI Ophthalmics, Pembroke, MA), Iris hooks (MST Inc., Redmond, WA) and retractors. All cases received standard topical and intra-cameral mydriatics.

### Data acquisition and subjects

Data was collected from the patient medical records system (Medisoft, Ltd, Leeds, UK) for cataract-alone surgery. Clinical variables were registered for age at surgery and gender, date of cataract surgery and laterality, DM status, the existence of PXF, the use of mechanical pupil expansion devices, and the incidence of postoperative uveitis and PCME after cataract surgery. All surgeries for patients under 18 years were excluded (N=105). Anti-inflammatory medication protocol did not differ in regard to the use of pupil expansion rings. All patients had the same follow-up for 4-6 weeks post operation. Post operative Uvetis was considered when a prolonged postoperative inflammation was observed using the slit-lamp examination, with characteristics of aqueous flare or cells, at 4-6 weeks following the operation. Pseudophakic cystoid macular edema (PCME) was diagnosed using the Macular OCT. All patients received the same post-operative topical treatment protocol with a mild variation of steroid topical eye drop frequency depending on the postoperative inflammation.

### Statistical analyses

Data are presented as mean ± standard deviation (SD) or absolute values and proportions. Statistical analysis was performed using IBM SPSS Statistics 27 (IBM SPSS Statistics for Windows, Version 27.0. IBM Corp., Armonk, NY). A two-factor Chi-square test was used for qualitative data and a Student’s t-test for continuous variables for two-group comparisons. Odds ratios for these postoperative complications when using pupil expansion devices were calculated using univariate regression analysis. Moreover, risk factors for PCME were assessed by multivariate regression analysis - having PCME as the dependent variable and age, gender, diabetes, pseudoexfoliation, and the use of pupil expansion device as independent variables. Kaplan-Meier curves were generated, and a log-rank test was used to assess Nd : YAG laser capsulotomy-free survival. Multivariate Cox regression controlling for age and gender was used to estimate hazard ratios (HR) for Nd : YAG laser capsulotomies. P-values less than 0.05 were considered statistically significant.

## Results

Included were 40,159 consecutive cataract surgeries. Overall, 39,460 eyes were operated without a pupil expansion device, and 699 were operated with the device. The mean age of patients operated on with and without pupil expansion devices was 77.7 ± 14.6 years and 79.1 ± 11.8 years, respectively (p=0.002, **Table 1**). The respective male: female ratios were 52.2:47.8% and 41.8:58.2% (p<0.001, **Table 1**). Furthermore, pseudoexfoliation syndrome was more frequently observed among eyes operated with pupil expansion devices than among those without the devices (p<0.001, **Table 1**). Laterality of the operated eyes and the presence of diabetes were not significantly different between the groups (**Table 1**).

**Table 1:** Baseline parameters according to the use of pupil expansion device.

	Pupil expansion device - N = 39460	Pupil expansion device + N = 699	P-value
Age (years)	79.1 ± 11.8	77.7 ± 14.6	**0.002**
Gender (male:female)	16503:22957 (41.8:58.2%)	365:334 (52.2:47.8%)	**<0.001**
Laterality (right:left)	20151:19309 (51.1:48.9%)	337:362 (48.2:51.8%)	0.134
DM	8107 (21.6%)	165 (24.6%)	0.057
PXF	87 (0.2%)	8 (1.2%)	**<0.001**

Data are given as mean (± SD) or absolute values (and proportions). DM, diabetes mellitus; PXF, pseudoexfoliation. For two-group comparisons, two-factor Chi-square test was used for qualitative data and Student’s t-test for continuous variables.Bold values are statistically significant.

### Risk of uveitis and pseudophakic cystoid macular edema after cataract surgery in eyes operated with and without pupil expansion devices

Postoperative uveitis without PCME was observed in 3.9% of the eyes operated with pupil expansion devices compared to 2.3% of those without the devices (p=0.005, **Table 2**). PCME was noted in 2.7% of the eyes operated with pupil expansion devices compared to 1.3% of those operated without the devices (p=0.002, **Table 2**). Postoperative uveitis or PCME, was reported in 6.6% of the eyes operated with pupil expansion devices compared to 3.6% of the eyes operated without the devices (p<0.001, **Table 2**).

**Table 2:** Clinical outcomes according to the use of pupil expansion device.

	Without expansion device N = 39460	With expansion device N = 699	P-value
PCME without postoperative uveitis	891 (2.3%)	27 (3.9%)	**0.005**
PCME with postoperative uveitis	528 (1.3%)	19 (2.7%)	**0.002**
PCME or postoperative uveitis	1419 (3.6%)	46 (6.6%)	**<0.001**

For two-group comparisons, two-factor Chi-square test was used. PCME, pseudophakic cystoid macular edema; w/o, without.Bold values are statistically significant.

In univariate regression analysis, eyes operated with pupil expansion devices showed higher odds ratios for postoperative uveitis without PMCE (OR 1.74, 95% CI 1.18-2.57, p=0.005, **Table 3**), for postoperative PCME (OR 2.06, 95% CI 1.30-3.28, p=0.002, **Table 3**), and postoperative uveitis or PMCE (OR 1.89, 95% CI 1.39-2.56, p<0.001, **Table 3**) after cataract surgery compared to eyes operated without the devices. In multivariate regression analysis, the risk for PMCE was greater in diabetic patients and eyes operated with pupil expansion devices than in those without the device (OR 1.50, 95% CI 1.24-1.83, p<0.001; OR 1.91, 95% CI 1.17-3.12, p=0.01, **Table 4**). The risk for PCME was lower among female patients when compared to men (OR 0.82, 95% CI 0.69-0.98, p=0.03, **Table 4**).

**Table 3:** Odds ratios for postoperative complications when using pupil expansion device.

	OR	95% CI	P-value
Postoperative uveitis w/o PCME	1.739	1.177 - 2.570	**0.005**
PCME	2.060	1.296 - 3.276	**0.002**
Postoperative uveitis or PCME	1.888	1.394 - 2.558	**<0.001**

Postoperative complications when using pupil expansion device (surgeries without pupil expansion device were used as cohort).Bold values are statistically significant.

**Table 4:** Multivariate regression analysis for PCME.

Variable	OR	95% CI	P-value
Age	1.004	0.996 - 1.012	0.388
Gender (female)	0.822	0.688 - 0.981	**0.030**
Diabetes	1.508	1.241 - 1.832	**<0.001**
PXF	1.667	0.408 - 6.810	0.477
Pupil expansion device	1.907	1.167 - 3.116	**0.010**

Logistic regression analysis having PCME as dependent variable and age, gender, diabetes, PXF and use of pupil expansion device as independent variables. PXF, pseudoexfoliation.Bold values are statistically significant.

### Risk of posterior capsule opacification after cataract surgery in eyes operated with and without pupil expansion device

**Figure 1:**
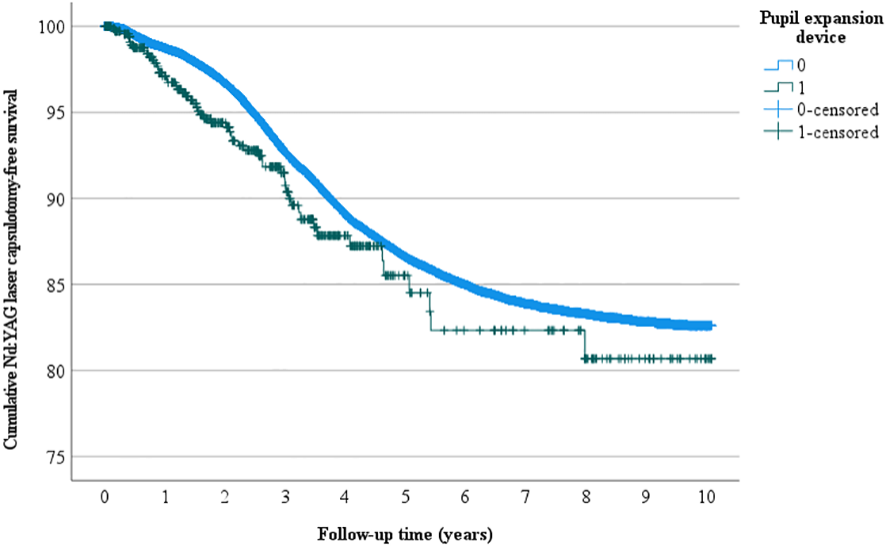
*Univariate Nd: YAG laser capsulotomy-free survival*. Univariate analysis of the cumulative incidence of Nd: YAG laser capsulotomy-free survival rate (%) during the follow-up (years) after surgery.

**Figure 2:**
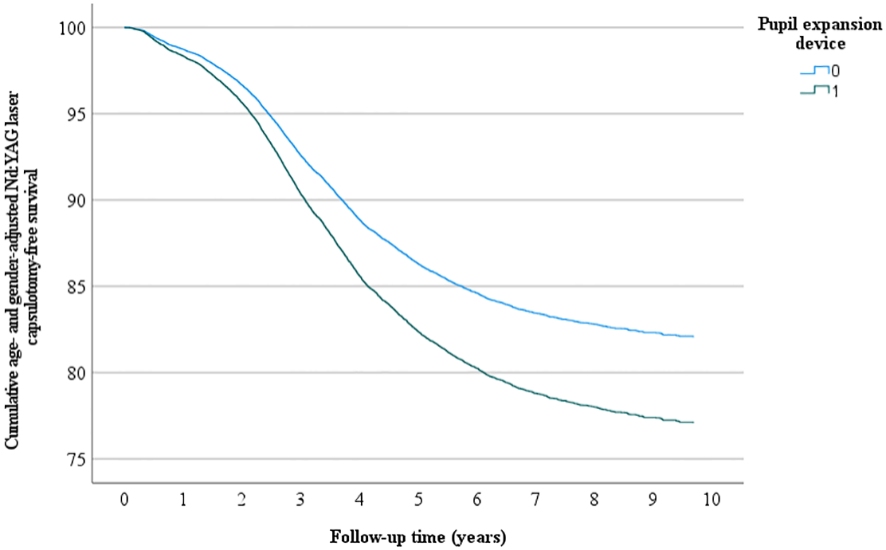
*Multivariate Nd: YAG laser capsulotomy- free survival*. Multivariate analysis - adjusted for age and gender - of the cumulative incidence of Nd: YAG laser capsulotomy-free survival rate (%) during the follow-up (years) after surgery.

Overall, 4522 eyes had Nd: YAG laser capsulotomy, 56 in the pupil expansion group and 4466 in the group without pupil expansion. Nd : YAG laser capsulotomy rates between the eyes operated with and without pupil expansion devices were compared by univariate Kaplan-Meier and multivariate Cox regression analyses. In univariate analysis, the eyes operated with pupil expansion devices tended to have higher Nd : YAG laser capsulotomy rates than those operated without the devices (p=0.053, **Figure 1**). In Cox regression analysis adjusted for the patients’ age and gender, the eyes operated with the devices had significantly higher Nd : YAG laser capsulotomy rates than those operated without the devices (HR 1.32, 95% CI 1.01-1.71, p=0.041, **Figure 2**).

## Discussion

This large real-world study of over 40,000 eyes demonstrated that eyes operated with pupil expansion devices had a higher risk for major postoperative complications after cataract surgery, namely uveitis, PMCE, and PCO.

Our results are supported by findings of earlier studies with a smaller number of patients that linked an increased risk of PCME to iris injury (6, 7). The use of a pupil expansion device (Malyugin ring^®^) was studied in a *post hoc* analysis of 536 eyes of 536 patients undergoing uneventful cataract surgery: 34 eyes with pupil expansion device and 502 eyes without the device. At one month, the eyes operated with the device exhibited increased aqueous flare and central subfield macular thickness increase compared to those without the device (6). During the 3-month follow-up, clinically significant PCME was documented in 12% of the eyes with pupil expansion devices and 2% without the device; the risk for PCME remained significant after confounders were included (6). In a prospective study on 98 eyes following cataract surgery, PCME occurred in 70.0% of patients with and 20.5% without iris trauma (7). Compared to individuals with less inflammation, those with postoperative anterior chamber inflammation of 2+ or more had a higher incidence of PCME (7).

The etiological factors leading to an increased rate of uveitis and PCME in eyes with pupil expansion devices may be related to inflammatory mediators generated in the anterior segment, which travel through the vitreous, enter the posterior segment and disrupt the blood-retinal barrier in the macular region, causing intraretinal fluid to accumulate (8). As the iris is a metabolically active tissue, pro-inflammatory cytokines (interleukin-6, prostaglandin E2, and tumor necrosis factor-alpha) are released following surgical injury, trauma, or ischemia (9–14). These cytokines ultimately result in a cascade of inflammation with clinical manifestations of uveitis or PCME (15, 16). Since our study emphasizes that using pupil expansion devices during cataract surgery increases the risk of clinically significant PCME, the surgeon should consider administering more potent anti-inflammatory drugs when planning postoperative management (17). This study also demonstrated that PXF occurrence was more common in eyes with pupil expansion devices. However, further investigation using multivariate regression analysis for PCME, as shown in **Table 4**, revealed no association.

Previously, we reported that IOL lens properties (i.e., dioptric power, biomechanics/haptic design) and the type of diabetes were associated with Nd : YAG laser capsulotomy rates (18–20). This study presents an increased rate of Nd : YAG laser capsulotomies, a surrogate marker for PCO, among eyes operated with pupil expansion devices. The proliferation of lens epithelial cells and the degree of postoperative inflammation are associated with the development of PCO. The inflammation causes epithelial cells to produce cytokines, which induce collagen production and fibrous metaplasia. The mechanisms include, e.g., activation and signaling of reactive oxygen species (ROS), nuclear factor kappa B (NF-κB) pathway, and pro-inflammatory and -fibrotic cytokines, namely tumor necrosis factor-alpha (TNFα) and transforming growth factor-beta (TGFβ) (21, 22). Jiang et al. reported that the LEC transcriptome was altered within 24 hours after cataract surgery, with most changes in the expression of genes regulating the innate immune responses (over 1000-fold upregulation) (23). Massive upregulation of inflammatory mediators (such as CXCL1, S100a9, CSF3, COX-2, CCL2, LCN2, and HMOX1) in LECs between 1 to 6 hours with their peak at 24 hours was followed by neutrophil infiltration (18 hours after surgery), TGFβ signaling upregulation (48 hours after surgery) and macrophage infiltration (3 days after surgery) (23). Furthermore, LECs produce interleukin-1, interleukin-6, basic fibroblast growth factor (bFGF), and fibronectin, which activate the transformation of LECs, proliferation, metaplasia around the equator of the anterior capsule, and migration toward the posterior capsule, leading to thickening and hypertrophy (24, 25). In addition, cytokines, such as epidermal growth factor (EGF), platelet-derived growth factor (PDGF), hepatocyte growth factor (HGF), and matrix metalloproteinases (MMPs) affect LEC activity (26). Both humoral and cellular immunity involvement after iris manipulation might explain the increased risk of PCO in eyes operated with pupil expansion devices.

Our study has some limitations. First, given its retrospective design, not all possible confounders could be accounted for or measured (as differences in surgical methods among surgeons). Second, the incidence and degree of PCO were not directly measured but were analyzed based on the need for Nd : YAG capsulotomy following surgery as an estimate of clinically significant PCO. Third, differences were observed in baseline characteristics between the groups, such as age and gender distribution. Furthermore, there was an expected difference in pseudoexfoliation syndrome; all these differences were considered in multivariate analysis. Fourth, in this study, diabetes was found to be an independent risk factor for PCME. However, we did not address the diabetic population who had a history of DME or diabetic retinopathy, both of which are recognized to be risk factors for the development of PCME (27, 28). Diabetes was also acknowledged as a confounder in the multivariate analysis. Another limitation is the lack of comparison of the various pupil expansion devices, which was beyond the scope of this study. Finally, there may be reporting bias as clinicians must select the presence of ocular pathologies and report the occurrence of surgical complications”.

In conclusion, our study demonstrates that using pupil expansion devices during cataract surgery may increase the risk for major postoperative complications and should be considered when planning the anti-inflammatory medication regime and follow-up after the surgery.

## Data availability statement

The original contributions presented in the study are included in the article/supplementary material. Further inquiries can be directed to the corresponding author.

## Ethics statement

The studies involving humans were approved by the local ethics community approval (CORN/SE/2021-2022/02 and was presented to the local audit authority) and adhered to the tenets of the Declaration of Helsinki. The studies were conducted in accordance with the local legislation and institutional requirements. Written informed consent for participation was not required from the participants or the participants’ legal guardians/next of kin in accordance with the national legislation and institutional requirements.

## Author contributions

TY: Formal Analysis, Writing – original draft. OE: Data curation, Writing – review & editing. VA: Data curation, Methodology, Writing – review & editing. DT: Formal Analysis, Investigation, Validation, Writing – review & editing. KD: Methodology, Writing – review & editing. EL: Methodology, Writing – review & editing. RT: Conceptualization, Data curation, Methodology, Supervision, Writing – review & editing. AA: Conceptualization, Data curation, Methodology, Supervision, Writing – review & editing.
